# Tongue-coating microbiota as a predictive biomarker of washed microbiota transplantation efficacy in pediatric autism: integration with clinical features

**DOI:** 10.1186/s12967-025-06846-z

**Published:** 2025-07-16

**Authors:** Hao-Jie Zhong, Zhao-Yu Pan, Yao-Fei Wei, Qian Yu, Lei Wu, Hong Wei, Xing-Xiang He

**Affiliations:** 1 Department of Gastroenterology, Research Center for Engineering Techniques of Microbiota-Targeted Therapies of Guangdong Province, The First Affiliated Hospital of Guangdong Pharmaceutical University, Guangzhou, China; 2 Department of Hepatobiliary and Pancreatic Surgery, Shenzhen Second People’s Hospital, The First Affiliated Hospital of Shenzhen University, Shenzhen, China; 3 The Department of Rheumatology, Shenzhen Traditional Chinese Medicine Hospital, The Fourth Clinical Medical College of Guangzhou University of Chinese Medicine, Shenzhen, China; 4 Precision Medicine Institute, The First Affiliated Hospital, Sun Yat-sen University, Guangzhou, China; 5 The Second Clinical Medical School of Guangzhou, University of Chinese Medicine, Guangzhou, China; 6 Yu-Yue Pathology Scientific Research Center, Chongqing, 401329 China; 7 Department of Gastroenterology, The First Affiliated Hospital of Guangdong Pharmaceutical University, Guangzhou, 510000 China

**Keywords:** Autism spectrum disorder, Fecal microbiota transplantation, Gut microbiota, Oral microbiota, Tongue-coating microbiota, Washed microbiota transplantation

## Abstract

**Background:**

Alterations in both oral and gut microbiota have been identified in children with autism spectrum disorder (ASD), but the interaction between these microbiota and their potential to predict outcomes of fecal microbiota transplantation (FMT) remain poorly understood.

**Methods:**

This study investigated the structure and function of the tongue-coating microbiota in children with ASD and explored its correlation with ASD symptoms and gut microbiota. Germ-free ASD mice, colonized with healthy gut microbiota, and children with ASD treated with washed microbiota transplantation (WMT) were assessed for changes in autism symptoms and microbiota composition. Predictive models were also developed based on pre-treatment tongue-coating microbiota and clinical features to forecast WMT outcomes.

**Results:**

Significant alterations were detected in the tongue-coating microbiota of children with ASD, with several bacterial species showing associations with ASD symptoms and gut microbiota composition. Following WMT, both mice and children exhibited substantial improvements in autism-related behaviors, alongside marked shifts in their gut and tongue-coating microbiota. A significant decrease in *Haemophilus* in the tongue-coating microbiota, which positively correlated with ASD severity, was observed. Additionally, a reduction in chemoheterotrophic and fermentation functions in the tongue-coating microbiota was identified. Predictive models utilizing pre-treatment tongue-coating microbiota and clinical data demonstrated comparable accuracy to those based on gut microbiota for forecasting WMT outcomes.

**Conclusions:**

These findings highlight a significant interaction between gut and tongue-coating microbiota in ASD, which may play a pivotal role in treatment outcomes. Predictive models integrating pre-treatment microbiota and clinical features could improve precision treatment strategies for children with ASD undergoing WMT.

**Graphical abstract:**

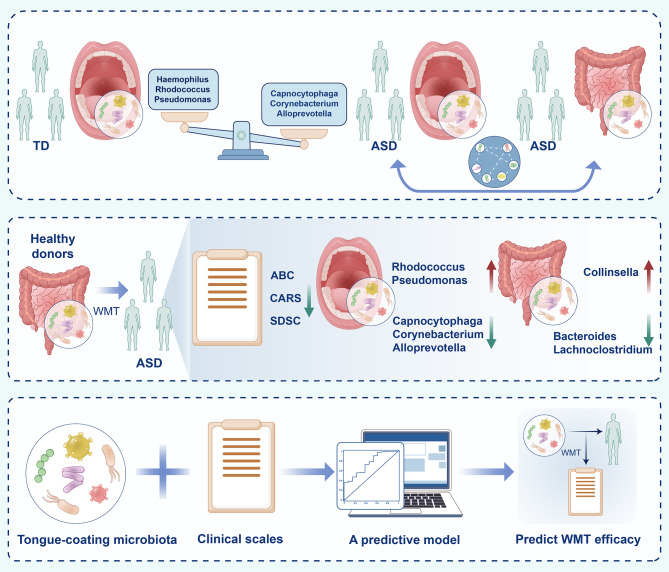

**Supplementary Information:**

The online version contains supplementary material available at 10.1186/s12967-025-06846-z.

## Background

Autism spectrum disorder (ASD) is a neurodevelopmental condition characterized by deficits in social communication and repetitive behaviors, impacting approximately 1% of children globally [1]. Despite incomplete understanding of its etiology, genetic, microbial, immune, and nutritional factors are recognized as contributors [2].

The gut microbiota, the body’s largest microbial community, has been linked to neuropsychiatric disorders, including ASD [3–5]. While some attribute gut microbial changes in ASD primarily to dietary preferences [6], accumulating evidence suggests a more active role of gut microbes in ASD pathogenesis [7, 8]. Fecal microbiota transplantation (FMT) holds promise for improving ASD symptoms by modulating gut microbiota [9–11], but therapeutic outcomes vary significantly among individuals. While baseline gut microbiota composition influences FMT efficacy, no predictive models currently exist to tailor treatment strategies [10].

Beyond the gut, the oral cavity hosts the second largest microbial community and serves as the entry point to the gastrointestinal tract [12]. Approximately 45% of oral taxa overlap with gut microbes and can migrate to and interact with the gut microbiota [13–15]. Studies have highlighted notable differences in oral microbiota between children with ASD and typically developing (TD) peers [16–18], with oral microbiota from ASD patients shown to induce autism-like behaviors in mice [19]. Recent research suggests that oral microbiota may also predict treatment outcomes [20, 21]. However, the interplay between oral and gut microbiota in ASD and their predictive value for therapeutic response remain underexplored.

In this study, significant structural and functional changes were identified in the tongue-coating microbiota of children with ASD, with several bacterial taxa correlating with both ASD symptoms and gut microbiota composition. Germ-free ASD mice colonized with healthy gut microbiota demonstrated behavioral improvements, reduced neuroinflammation, and alterations in tongue-coating microbiota, in contrast to those receiving ASD-associated microbiota. Clinically, washed microbiota transplantation (WMT), an optimized version of FMT designed to reduce adverse reactions by repeatedly washing bacteria, effectively alleviated ASD symptoms and reshaped the tongue-coating microbiota toward a TD-like profile. Furthermore, certain pre-treatment tongue-coating bacterial species were linked to WMT response. A predictive model combining tongue-coating microbiota with clinical features accurately forecasted WMT efficacy, performing similarly to models based on gut microbiota. These findings underscore the oral-gut microbial axis in ASD and suggest that tongue-coating microbiota, in conjunction with clinical indicators, could serve as a practical, non-invasive tool for predicting microbiota-based treatment outcomes.

## Methods

### Study design and ethics

This single-center, retrospective cohort study was conducted at the First Affiliated Hospital of Guangdong Pharmaceutical University in Guangzhou, China. Ethical approval was granted by the Ethics Committee of the First Affiliated Hospital of Guangdong Pharmaceutical University (No. 2020-14), and written informed consent was obtained from the parents or guardians of all participating children.

### Participants

Children who were admitted for WMT at the hospital between June 2019 and October 2022 were eligible for inclusion. Exclusion criteria included antibiotic use within one month prior to WMT, severe dental conditions (e.g., gingivitis with bleeding at more than 10 probing sites), history of major systemic diseases (e.g., significant heart, lung, or kidney issues), or lack of follow-up data after WMT. ASD diagnoses were made according to the criteria outlined in the Diagnostic and Statistical Manual of Mental Disorders, Fifth Edition [22].

### Data collection

Demographic data, body mass index, duration of ASD, severity of core ASD symptoms, and sleep quality were extracted from the medical records. Core ASD symptoms were evaluated using the Childhood Autism Rating Scale (CARS) and the Aberrant Behavior Checklist (ABC) at baseline and during each follow-up visit [23, 24]. The CARS, a 15-item scale assessing communication, social interaction, emotional responses, and behavioral patterns, was administered by a pediatrician. The ABC, consisting of 58 items across five subscales (irritability, agitation, crying, lethargy, and stereotypic behavior), was completed by the child’s parents. Additionally, parents filled out the Sleep Disturbance Scale for Children (SDSC), a questionnaire evaluating sleep disturbances [25]. Children demonstrating significant improvement in both ABC and CARS scores, with reductions ranking in the top 25% of responses, were classified as having an excellent treatment response.

### Sample collection

Tongue-coating and fecal samples were collected from children with ASD prior to each WMT treatment. For tongue-coating sample collection, patients were instructed to refrain from eating or brushing their teeth for at least one hour before sampling. Samples were obtained between 9:00 AM and 12:00 PM using sterile cotton swabs from the middle of the tongue, placed into RNase-free Eppendorf tubes, and then 1 mL of PBS was added within ten minutes of collection. After gentle agitation to release bacteria into the PBS solution, the bacterial suspension was centrifuged at 4000 rpm for 25 min. The supernatant was discarded, and the remaining sediment was stored at -80 °C for subsequent sequencing analysis. For comparison, tongue-coating samples were also collected from 35 TD children. For fecal sample collection, parents were instructed to collect a portion of stool within ten minutes after defecation, ensuring the sample did not come into contact with urine or toilet water. Fecal samples were then transferred to fecal DNA preservation tubes (Invitek, Germany) and stored at -80 °C until further processing.

### Washed microbiota transplantation

Donor screening [11] and the preparation of the washed microbiota suspension [26] were conducted as previously described. Briefly, donors underwent an initial screening *via* a questionnaire, followed by blood and stool tests to exclude infectious diseases. A total of 25 individuals (11 males, 14 females; median age 25.0 years [interquartile range: 23.0–26.5]; mean BMI: 20.1 ± 2.1 kg/m^2^), all under the age of 30, with a BMI below 24, regular lifestyles, healthy dietary habits, normal bowel movements, no history of chronic or infectious diseases, and no recent antibiotic use, were enrolled as healthy donors. To prepare the microbiota suspension, 100 g of stool were homogenized in 500 mL of 0.9% saline and processed using an automated microbiota purification system (GenFMTer; FMT Medical, Nanjing, China) with microfiltration. The bacterial precipitate obtained after centrifugation was washed three times with saline. Finally, 100 mL of saline was added to resuspend the bacterial precipitate, resulting in the final washed microbiota suspension.

For WMT, children with ASD received 60 to 90 mL of the washed microbiota suspension *via* a transendoscopic enteral tube. Each treatment course consisted of consecutive bacterial transplantations for six days, with a one-month interval between courses. No antibiotics were administered prior to WMT. Donor microbiota was randomly selected from screened healthy individuals. Following WMT, patients were advised to adhere to a semi-liquid or soft diet, avoiding spicy foods, seafood, and other common dietary allergens.

### Germ-free mouse model

Shank3 knockout mice were obtained from GemPharmatech Co., Ltd., and genotyping was performed to confirm successful gene deletion before experimental use. The mice were rederived to germ-free status and housed in sterile isolators with ad libitum access to autoclaved food and water. To assess the direct effects of gut microbiota on ASD-related behaviors, neuroinflammation, intestinal pathology, and microbial composition in both the gut and oral cavity, eight-week-old male germ-free Shank3 knockout mice were randomly assigned to receive fecal microbiota from either an ASD donor or TD donors. FMT was performed twice weekly by oral gavage. Specifically, 1 g of donor feces was homogenized in 10 mL sterile PBS, filtered to remove particulate matter, and 200 µL of the resulting suspension was administered to each mouse. Behavioral assessments were conducted, and mice were sacrificed at week 50 post-gavage. All procedures were approved by the Independent Ethics Committee for Clinical Research and Animal Trials of the First Affiliated Hospital of Sun Yat-sen University (Approval No. 2023 − 157).

### Three-chamber social test

A test to evaluate sociability in mice was conducted using a three-chambered apparatus [27]. After 5 min of habituation, an unfamiliar mouse was placed in a wire cage in one side chamber, and an empty cage or toy was placed in the other chamber. The test mouse was allowed to explore for 10 min. The time spent in each chamber and the interaction time with the unfamiliar mouse versus the object were recorded to assess social behavior.

### Grooming test

The grooming test was conducted to assess repetitive behaviors as previously described [27]. Prior to the test, each mouse was placed in a clean, empty cage for five minutes of habituation, followed by a ten-minute recording period. Grooming time was measured by a trained observer.

### Flow cytometry

Leukocytes from brain tissues were isolated using mechanical dissociation followed by Percoll density gradient centrifugation, as previously described [28]. Peripheral blood mononuclear cell (PBMC)-derived leukocytes were obtained using ACK lysis buffer. To characterize Th17, Treg, M1, and M2 subsets, surface staining was performed using antibodies against CD3 (BioLegend, 100204), CD4 (BioLegend, 100528), CD11b (BioLegend, 101257), F4/80 (BioLegend, 123132), CD86 (BioLegend, 105036), and CD206 (BioLegend, 141734). Intracellular staining for RORγt (Invitrogen, 17-6988-82) and Foxp3 (BioLegend, 320008) was performed following fixation and permeabilization with the Foxp3 Transcription Factor Staining Kit (eBioscience), in accordance with the manufacturer’s instructions. Flow cytometric analysis was carried out using a CytoFLEX flow cytometer (Beckman Coulter).

### Enzyme-linked immunosorbent assay (ELISA)

The concentrations of TNF-α, IL-1β, and IL-6 in mouse tissues were quantified using ELISA kits (BioLegend; catalog numbers: 430901, 432604, and 431304), following the manufacturer’s protocols.

### Histological analysis

After overnight fixation in 4% paraformaldehyde, the small intestines of mice were dehydrated, paraffin-embedded, and sectioned into 2 μm slices. These sections were stained with hematoxylin and eosin, and villous height and crypt depth were randomly measured for three well-oriented villi of the small intestine per mouse.

### DNA extraction and 16S rRNA gene sequencing

Total DNA was extracted using the E.Z.N.A.^®^ Soil DNA Kit (Omega Bio-tek, Norcross, GA, USA) according to the manufacturer’s instructions. Bacterial 16S rRNA gene fragments (V3-V4) were amplified from the extracted DNA using primers 338 F (5’-ACTCCTACGGGAGGCAGCAG-3’) and 806R (5’-GGACTACHVGGGTWTCTAAT-3’). The amplicon size was confirmed through agarose gel electrophoresis. After confirmation, paired-end sequencing was performed on the Illumina MiSeq platform using PE300 chemistry at Majorbio Bio-Pharm Technology Co. Ltd. (Shanghai, China).

### Bioinformatic analysis

The read pairs underwent demultiplexing, merging using FLASH (version 1.2.11) [29], and subsequent filtering with fastp (version 0.19.6) [30]. Reads shorter than 10 bp and those with a base quality score below Phred 20 were discarded. Paired reads with a minimum overlap of 10 bp were merged into single sequences, applying a maximum mismatch ratio of 0.2 to exclude non-conforming sequences. Samples were distinguished, and sequence orientation was adjusted based on the barcodes and primers located at both ends of the sequence. A strict tolerance for barcode mismatches was set at 0, while a threshold of up to 2 primer mismatches was allowed. High-quality sequences were de-noised using the DADA2 plugin [31] in the QIIME2 (version 2020.2) [32] pipeline with default parameters to obtain amplicon sequence variants (ASVs). Taxonomic assignment of the ASVs was conducted using the SILVA reference database. Microbial data analysis was performed using the Majorbio Cloud Platform (www.majorbio.com).

### Metabolomics analysis

Tissue samples (approximately 50 mg) were extracted using 400 µL of methanol (4:1, v/v) containing 0.02 mg/mL of L-2-chlorophenylalanine as an internal standard. The samples were homogenized at − 10 °C, ultrasonicated at 5 °C, and subjected to protein precipitation at − 20 °C, followed by centrifugation at 13,000 g for 15 min at 4 °C. Supernatants were collected for LC-MS/MS analysis. Pooled quality control samples were prepared by combining equal aliquots from all individual samples.

LC-MS/MS analysis was performed using a Thermo UHPLC-Q Exactive HF-X system with an ACQUITY HSS T3 column. The mobile phases consisted of 0.1% formic acid in water and a mixture of acetonitrile: isopropanol (47.5:47.5, v/v). The flow rate was set to 0.4 mL/min, the column temperature was maintained at 40 °C, and the injection volume was 3 µL. Data were acquired in both positive and negative ion modes using data-dependent acquisition, with MS and MS/MS resolutions set at 60,000 and 7,500, respectively.

Data processing was carried out using Progenesis QI for baseline correction, peak alignment, and normalization. Metabolite annotation was performed based on the HMDB, Metlin, and Majorbio databases, with statistical analyses conducted using the Majorbio cloud platform.

### Statistical analyses

Statistical analyses were performed using Prism 7.0 (GraphPad Prism). Continuous variables were expressed as mean ± standard deviation or median with interquartile ranges, and categorical variables were represented as counts and percentages. Group comparisons were made using the appropriate statistical tests, such as Student’s t-tests (paired or unpaired), Mann-Whitney rank sum tests, or Chi-square tests. Statistical significance was defined by a two-tailed *P*-value of < 0.05.

## Results

### The tongue-coating microbiota in children with ASD undergoes significant alterations and is closely associated with the gut microbiota

**Fig. 1:**
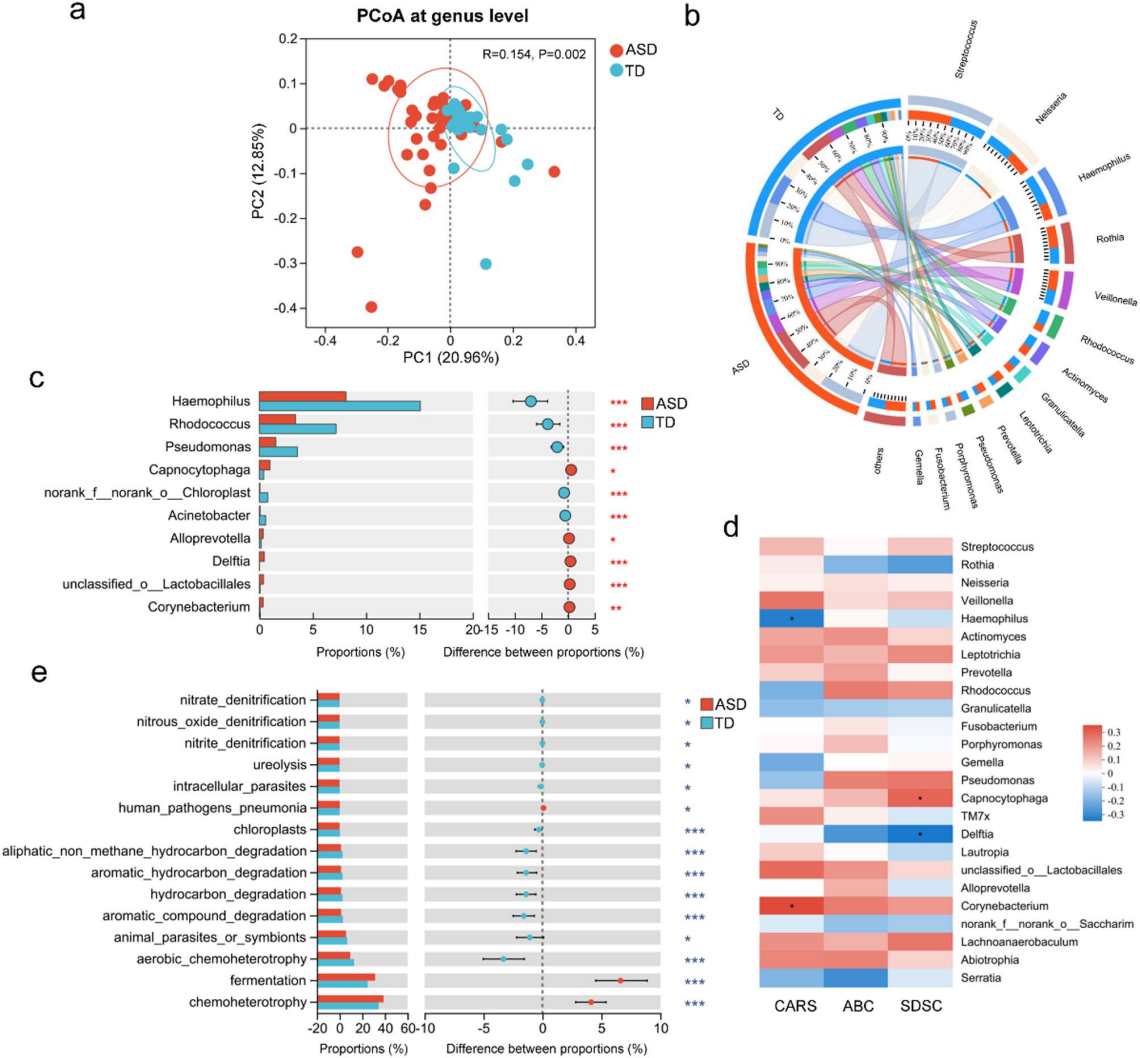
Children with autism spectrum disorders (ASD) exhibit altered tongue-coating microbiota structure and function. (**a**) Principal Coordinate Analysis (PCoA) comparing tongue-coating microbiota in children with ASD (*n* = 51) and typically developing (TD, *n* = 35) children. (**b**) Circos plot illustrating microbial composition at the genus level. (**c**) Bar chart displaying the relative abundances of the top 10 differentially abundant genera between ASD and TD children. (**d**) Heatmap showing the correlations between the abundance of genera and the severity of clinical symptoms in children with ASD. (**e**) Functional prediction analysis of tongue-coating microbiota based on FAPROTAX. ABC, Aberrant Behavior Checklist; CARS, Childhood Autism Rating Scale; SDSC, Sleep Disturbance Scale for Children. *p < 0.05; **p < 0.01; ***p < 0.001

To characterize the tongue-coating microbiota in children with ASD, 16S rRNA gene sequencing was performed on samples from 51 children with ASD and 35 TD children (age: 6.00 [4.00–7.00] vs. 6.00 [4.00–7.00], *p* = 0.702; male: 94.12% vs. 91.43%, *p* = 0.684). Alpha diversity analysis revealed significantly higher microbial richness in the ASD group, as evidenced by the Ace and Chao indices, while the Shannon index showed similar diversity between the groups (Additional file 1: Figure S1a). Principal coordinate analysis (PCoA) based on Jaccard distance demonstrated distinct microbial community structures between the ASD and TD groups (Fig. 1a). At the genus level, children with ASD showed a significant reduction in *Haemophilus*, *Rhodococcus*, and *Pseudomonas*, along with a notable increase in *Capnocytophaga*, *Alloprevotella*, and *Corynebacterium* compared to TD controls (Fig. 1b–c; Additional file 1: Figure S1b–d). Correlation analysis revealed negative associations between *Haemophilus* and CARS scores, and *Delftia* and SDSC scores, while *Corynebacterium* and Capn*o*cytophaga showed positive correlations with CARS and SDSC scores, respectively (Fig. 1d). Functional prediction using the FAPROTAX database indicated that the tongue-coating microbiota in the ASD group was enriched in functions related to chemoheterotrophy and fermentation (Fig. 1e).

Given the marked alterations in both oral and gut microbiota in children with ASD [17, 18], their associations were examined using a correlation network. Several tongue-coating genera, including *Capnocytophaga*, *Delftia*, and *Corynebacterium*—significantly associated with clinical scores—also displayed strong correlations with gut bacteria (correlation coefficients > 0.4; Additional file 2: Figure S2). These results highlight substantial structural and functional changes in the oral microbiota and suggest a strong oral–gut microbial interaction in ASD.

### A healthy gut microbiota can alleviate autism symptoms in both autistic mice and children

**Fig. 2:**
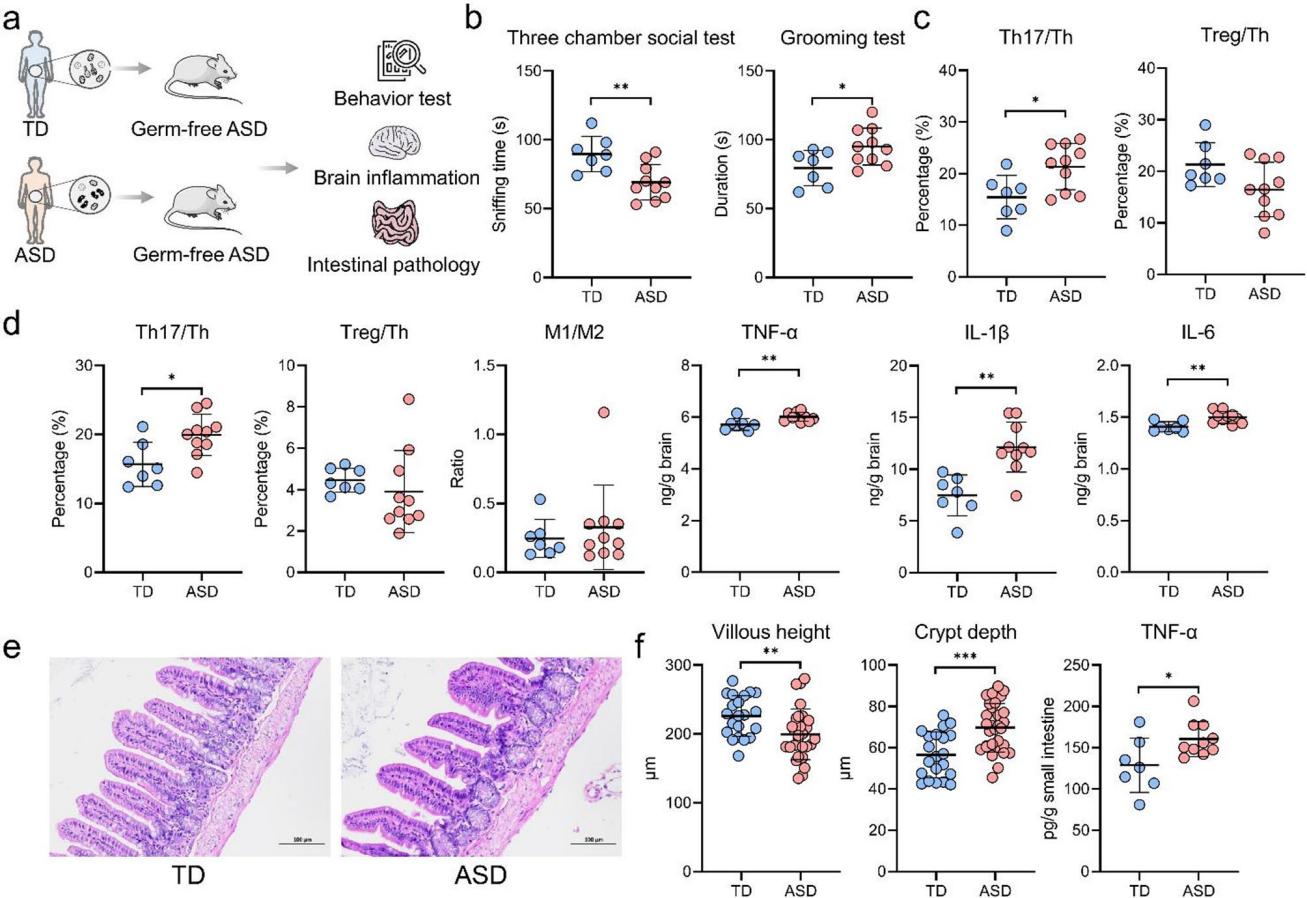
Transplantation of healthy gut microbiota alleviates autism-like behaviors, neuroinflammation, and intestinal pathology in mice with autism spectrum disorders (ASD). (**a**) Experimental design illustrating ASD mice receiving gut microbiota transplantation from typically developing (TD) children (*n* = 7) or from a child with ASD (*n* = 10). (**b**) Behavioral assessments including the three-chamber social interaction test and the self-grooming test. (**c**) Proportions of Th17 and Treg cells in peripheral blood. (**d**) Proportions of Th17 and Treg cells, M1/M2 macrophage ratio, and concentrations of TNF-α, IL-1β, and IL-6 in the brain. (**e**) Representative histological images of the small intestine. (**f**) Quantification of villus height, crypt depth, and TNF-α levels in the small intestine. *p < 0.05; **p < 0.01; ***p < 0.001

Transplanting gut microbiota from patients with ASD into germ-free mice induces autism-like behaviors [7, 33], but the effects of healthy gut microbiota on ASD mice remain inadequately understood. To investigate this, gut microbiota from TD children and children with ASD were transferred into germ-free ASD mice (Fig. 2a). Mice receiving healthy gut microbiota demonstrated significantly improved social interactions, reduced repetitive behaviors, lower Th17 cell levels in both blood and brain, and elevated levels of pro-inflammatory cytokines in the brain (Fig. 2b-d). Additionally, these mice exhibited increased intestinal villous height, reduced crypt depth, and decreased intestinal TNF-alpha levels (Fig. 2e-f). These results suggest that healthy gut microbiota can ameliorate core autistic behaviors, alleviate brain inflammation, and improve intestinal barrier function in ASD mice.

**Fig. 3:**
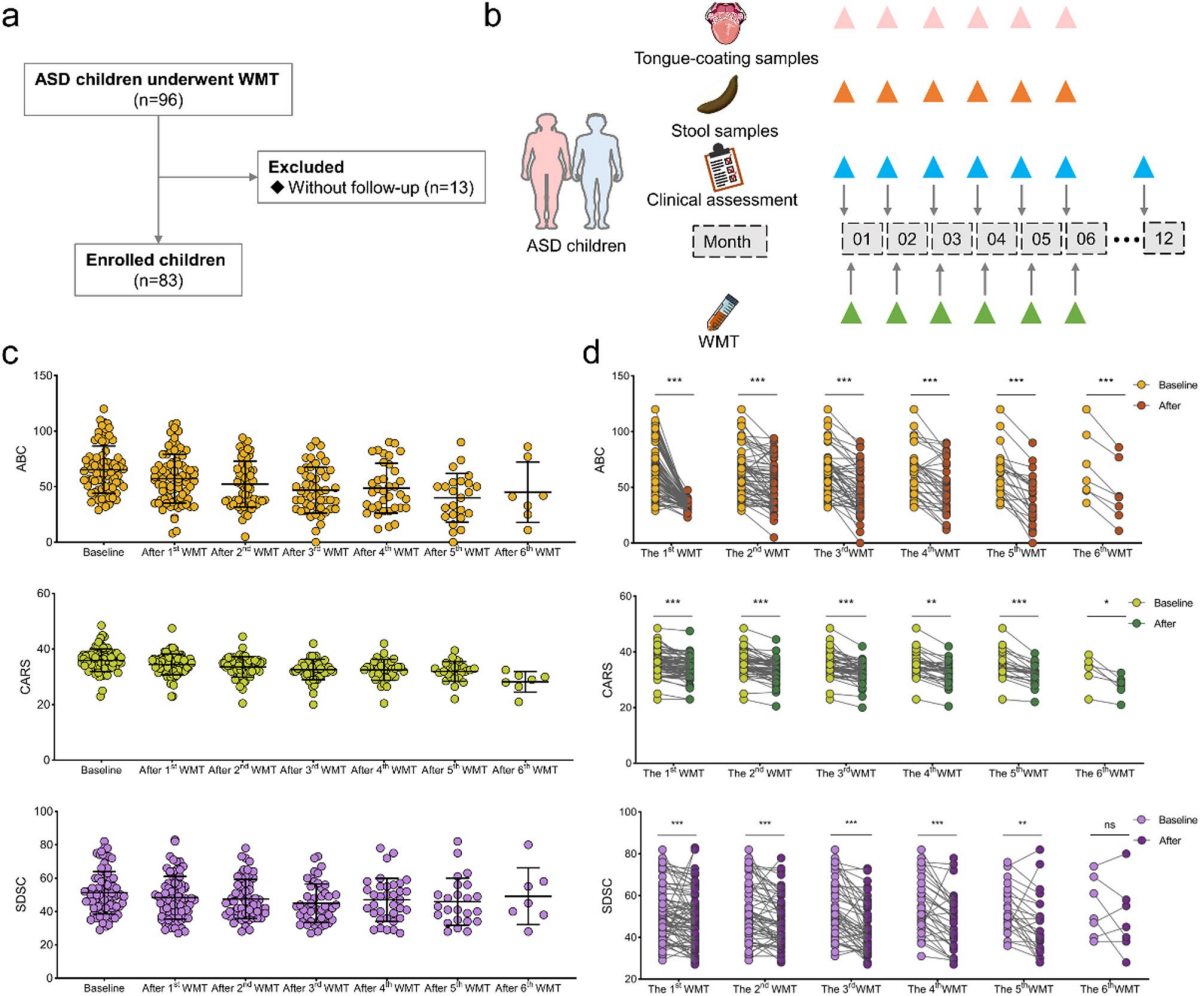
Washed microbiota transplantation (WMT) significantly alleviates autism symptoms and improves sleep quality in children with autism spectrum disorders (ASD). (**a**) Flow diagram of patient enrollment (*n* = 83). (**b**) Diagram of the study design. (**c**) Bar charts showing baseline and post-treatment levels of ABC (Aberrant Behavior Checklist), CARS (Childhood Autism Rating Scale), and SDSC (Sleep Disturbance Scale for Children) in children with ASD, measured after each course of WMT. (**d**) Comparative analysis of ABC, CARS, and SDSC scores in children with ASD, post-WMT treatment versus baseline. *p < 0.05; **p < 0.01; ***p < 0.001

To further assess the therapeutic impact of healthy gut microbiota on ASD, clinical data from 83 children with ASD who underwent WMT and subsequent follow-ups were analyzed (Fig. 3a-b, Additional file 7: Table S1). Of these, 19 children completed one WMT course, 14 completed two, 11 completed three, 14 completed four, 18 completed five, and 7 completed all six courses. Among the participants, 50 children underwent WMT in conjunction with other therapies (including behavioral or pharmacological treatments), while 33 received only WMT. With an increasing number of WMT courses, ABC, CARS, and SDSC scores gradually decreased (Fig. 3c). Significant reductions in CARS, ABC, and SDSC scores were observed after each WMT course compared to baseline, except for the SDSC scores following the sixth course (Fig. 3d). Seven adverse events were reported across 268 WMT treatment courses, primarily involving mild symptoms such as fever, diarrhea, abdominal pain, and vomiting. All symptoms were self-limited or resolved with symptomatic treatment, and no serious adverse events occurred. These results support the safety and potential efficacy of WMT as an intervention for improving ASD symptoms in children.

### Healthy gut microbiota transplantation potentially treats ASD by regulating the gut microbiota in both mice and children

**Fig. 4:**
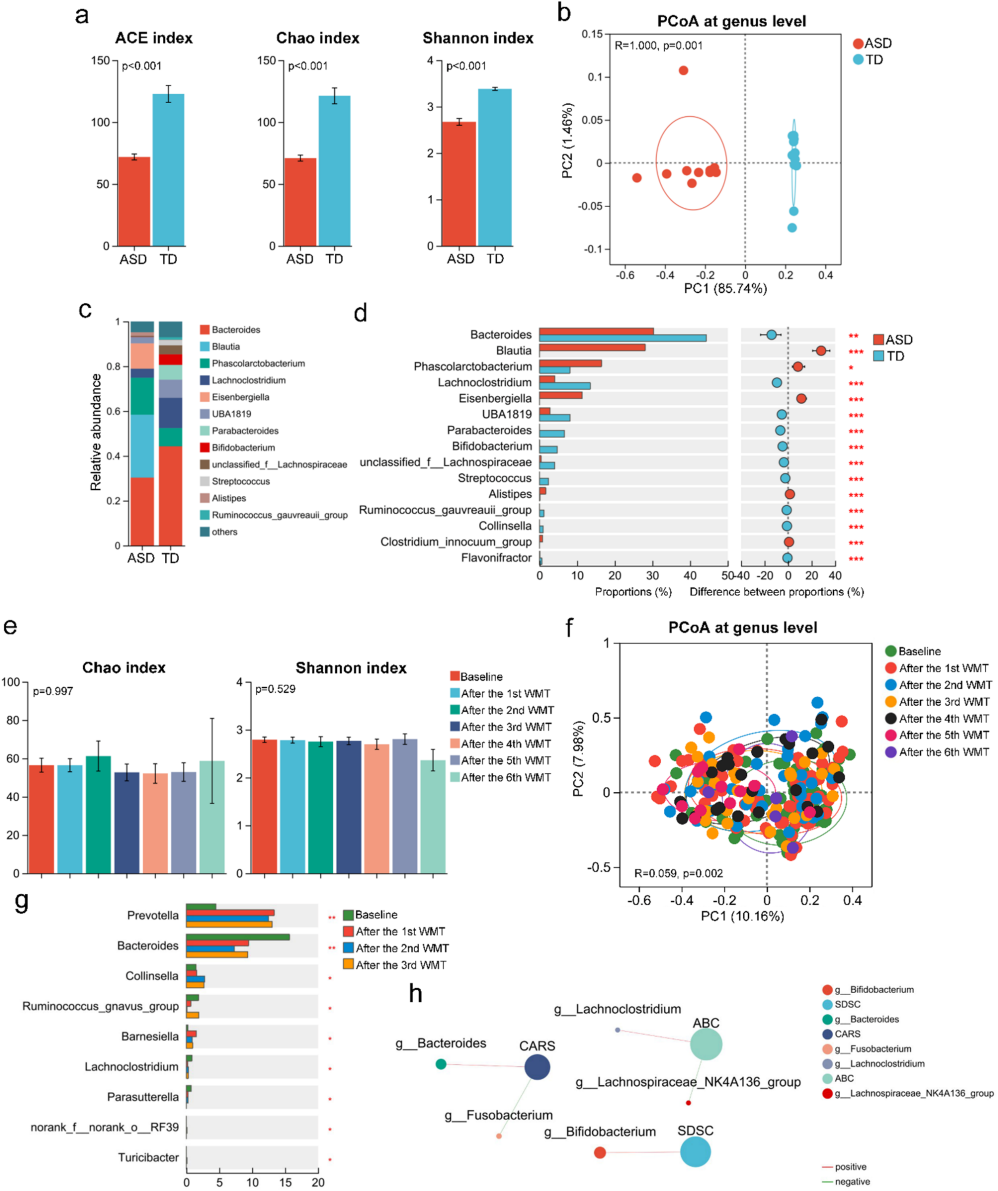
Effect of microbiota transplantation on the gut microbiota of mice and children with autism spectrum disorder (ASD). (**a**) Alpha diversity indices (Ace, Chao, Shannon) of gut microbiota in ASD mice transplanted with microbiota from an ASD child (*n* = 10) or TD children (*n* = 7). (**b**) Principal Coordinate Analysis (PCoA) showing structural differences in gut microbiota between the two mouse groups. (**c**) Genus-level taxonomic composition of gut microbiota in ASD mice. (**d**) Relative abundances of the top 15 differentially abundant genera in ASD mice. (**e**) Alpha diversity indices (Chao and Shannon) of gut microbiota in children with ASD (*n* = 72) before and after each WMT course. (**f**) PCoA showing structural shifts in gut microbiota of children with ASD across WMT courses. (**g**) Relative abundances of the top 15 differentially abundant genera in children with ASD before and after WMT. (**h**) Correlation analysis between gut microbiota and clinical scores in children with ASD (absolute correlation coefficients > 0.2). *p < 0.05; **p < 0.01

To characterize the gut microbiota in ASD mice following FMT, fecal samples were collected for 16S rRNA gene sequencing. Mice receiving healthy gut microbiota exhibited significantly increased richness and diversity in their gut microbiota compared to those receiving ASD microbiota (Fig. 4a). PCoA and genus abundance analyses revealed distinct differences in the structure and composition of the gut microbiota between the two groups (Fig. 4b-c). Further analysis revealed that mice with ASD microbiota had significantly lower levels of beneficial probiotics, such as *Lachnoclostridium*, *ParaBacteroides*, and *Bifidobacterium*, compared to those with microbiota from TD children (Fig. 4d). Additionally, the abundance of various intestinal bacteria correlated with ASD symptoms, central inflammation, and intestinal barrier function in these mice (Additional file 3: Figure S3). Notably, the concentration of 3-(3-sulfooxyphenyl) propanoic acid—a putative downstream metabolite of m-tyrosine—was significantly reduced in the gut, plasma, and brain of ASD mice following healthy microbiota transplantation (Additional file 8: Table S2). This compound, a structural analog of tyrosine, is known to deplete central catecholamines and induce autism-like behaviors in animal models [34]. However, the specific gut microbes responsible for its synthesis and its mechanistic role in ASD pathophysiology remain unclear.

To assess the effects of WMT on the gut microbiota of children with ASD, fecal samples were analyzed from children with ASD before and after WMT, as well as from WMT donors, using 16S rRNA gene sequencing. The characteristics of the donor gut microbiota are outlined in Additional file 4: Figure S4. Despite no significant changes in the richness and diversity of the gut microbiota post-WMT, PCoA revealed significant structural changes in the microbiota of children with ASD (Fig. 4e-f). Differential microbiota analysis indicated that children with ASD post-WMT showed a significant decrease in the abundance of *Bacteroides* and *Lachnoclostridium*, which were positively correlated with the CARS and ABC scales, respectively (Fig. 4g-h). Conversely, there was a significant increase in *Collinsella* abundance, a result consistent with observations in ASD mice receiving healthy gut microbiota transplants (Fig. 4g). These results suggest that WMT may provide therapeutic benefits by modulating the gut microbiota.

### Alterations in the tongue-coating microbiota after healthy microbiota transplantation in ASD mice and children suggest an interaction between gut and tongue microbiota

**Fig. 5:**
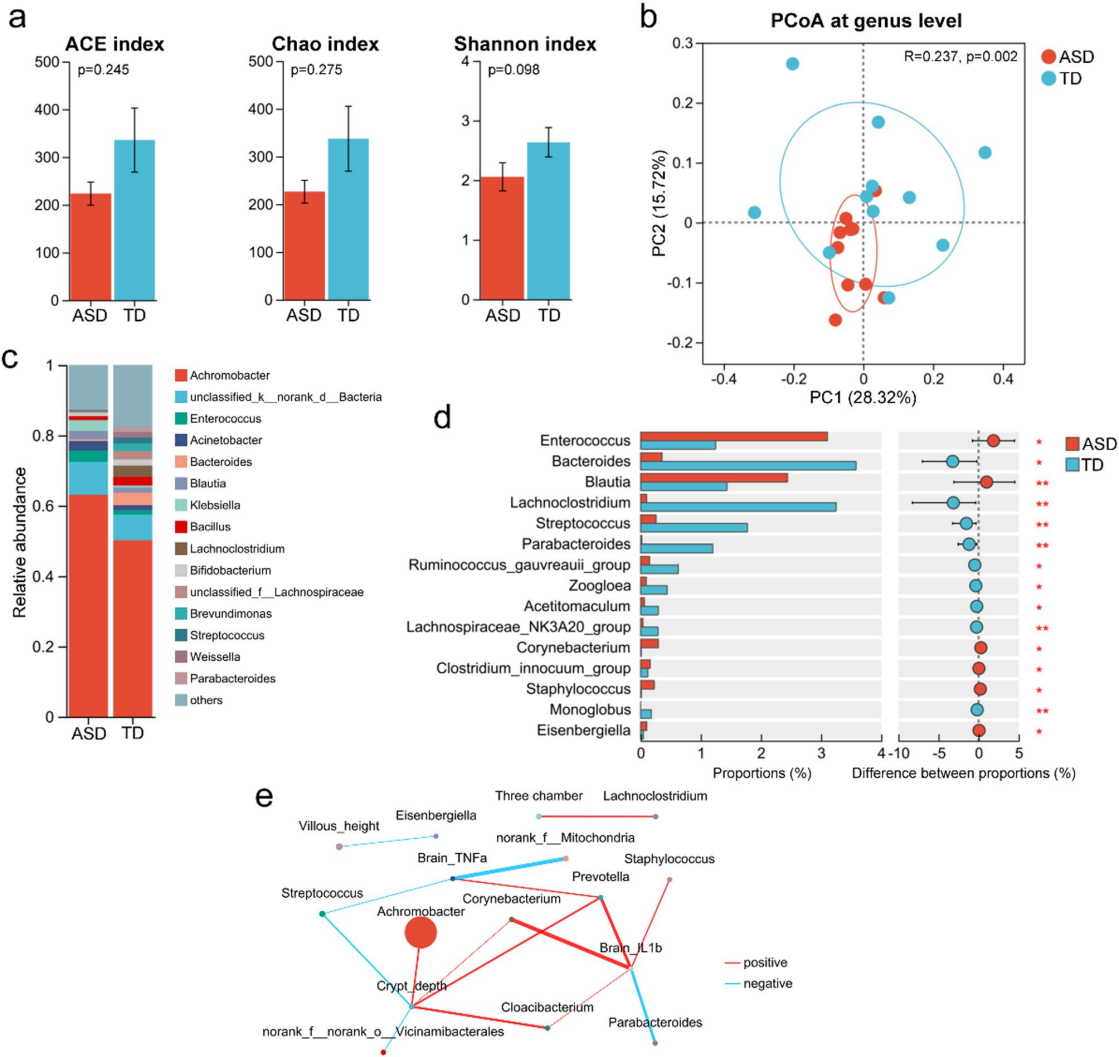
Transplantation of healthy gut microbiota significantly affects the tongue-coating microbiota structure in mice with autism spectrum disorders (ASD). (**a**) Alpha diversity indices (Ace, Chao, Shannon) of tongue-coating microbiota in ASD mice transplanted with microbiota from an ASD child (*n* = 10) or TD children (*n* = 11). (**b**) PCoA showing structural differences in tongue-coating microbiota between the two groups. (**c**) Genus-level taxonomic composition of tongue-coating microbiota. (**d**) Relative abundances of the top 15 differentially abundant genera. (**e**) Correlation analysis between tongue-coating microbiota and autism-like behaviors, neuroinflammatory markers, and intestinal pathology (absolute correlation coefficients > 0.6). *p < 0.05; **p < 0.01

Despite reported correlations between the oral and gut microbiota in patients with ASD, the interactive relationship between these microbiota remains poorly understood [18]. To explore this interaction, the tongue-coating microbiota of ASD mice following transplantation with either healthy or ASD gut microbiota was analyzed. No significant differences were observed in the Ace, Chao, and Shannon indices between the two groups, yet PCoA revealed distinct structural differences in their tongue-coating microbiota (Fig. 5a-b). Bar graph analysis indicated considerable variations in genus composition (Fig. 5c). Consistent with gut microbiota findings, mice transplanted with ASD gut microbiota displayed significantly reduced abundances of beneficial microbes such as *Lachnoclostridium* and *ParaBacteroides* in their tongue coatings, while levels of *Enterococcus*, *Blautia*, and *Staphylococcus* were notably elevated (Fig. 5d). Additionally, network analysis revealed correlations between the abundance of various tongue-coating bacteria and autism-like behaviors, neuroinflammatory markers, and intestinal pathology in ASD mice (Fig. 5e).

**Fig. 6:**
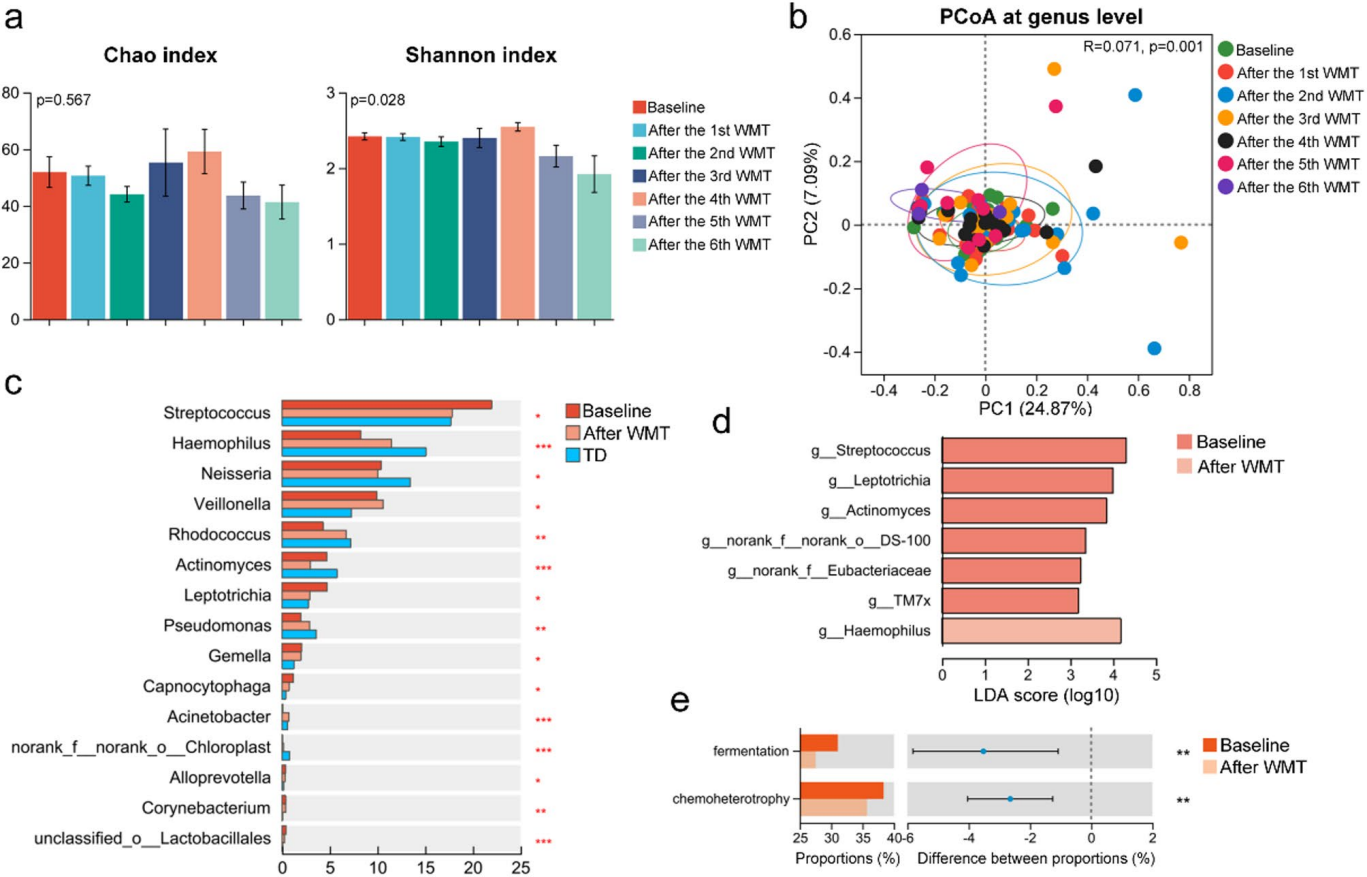
Washed microbiota transplantation (WMT) significantly alters the tongue-coating microbiota structure and function in children with autism spectrum disorders (ASD). (**a**) Alpha diversity indices (Chao and Shannon) of tongue-coating microbiota in children (*n* = 40) with ASD before and after each WMT course. (**b**) PCoA showing structural shifts in tongue-coating microbiota across WMT courses. (**c**) Relative abundances of the top 15 differentially abundant genera in children with ASD (before and after WMT) and in typically developing (TD) children. (**d**) LEfSe analysis identifying differentially enriched taxa (LDA score > 3) in children with ASD before and after WMT. (**e**) Predicted functional profiles of tongue-coating microbiota based on FAPROTAX before and after WMT. *p < 0.05; **p < 0.01; ***p < 0.001

Similarly, tongue-coating samples from children with ASD were analyzed before and after WMT. Alpha diversity analyses showed no significant changes in the Chao index post-WMT, though the Shannon index significantly decreased after the fifth and sixth WMT courses (Fig. 6a). PCoA also demonstrated significant structural alterations in the tongue-coating microbiota of children with ASD post-WMT (Fig. 6b). Increases in *Rhodococcus* and *Pseudomonas* were observed post-WMT, while *Capnocytophaga*, *Alloprevotella*, and *Corynebacterium* decreased, bringing their levels closer to those found in TD children (Fig. 6c). Notably, the significantly reduced abundance of *Haemophilus* in the tongue-coating of children with ASD, which negatively correlated with the CARS scale, markedly increased following WMT (Fig. 6c-d). Functional predictions of the microbiota revealed enhanced capabilities for chemoheterotrophy and fermentation in the tongue-coating microbiota of children with ASD, which were significantly reduced after WMT treatment (Fig. 6e). These results suggest that WMT significantly impacts both the structure and function of the tongue-coating microbiota in children with ASD, highlighting a potential interaction between the gut and tongue-coating microbiota.

### Pre-treatment gut microbiota combined with clinical characteristics can predict the efficacy of WMT in treating ASD in children

**Fig. 7:**
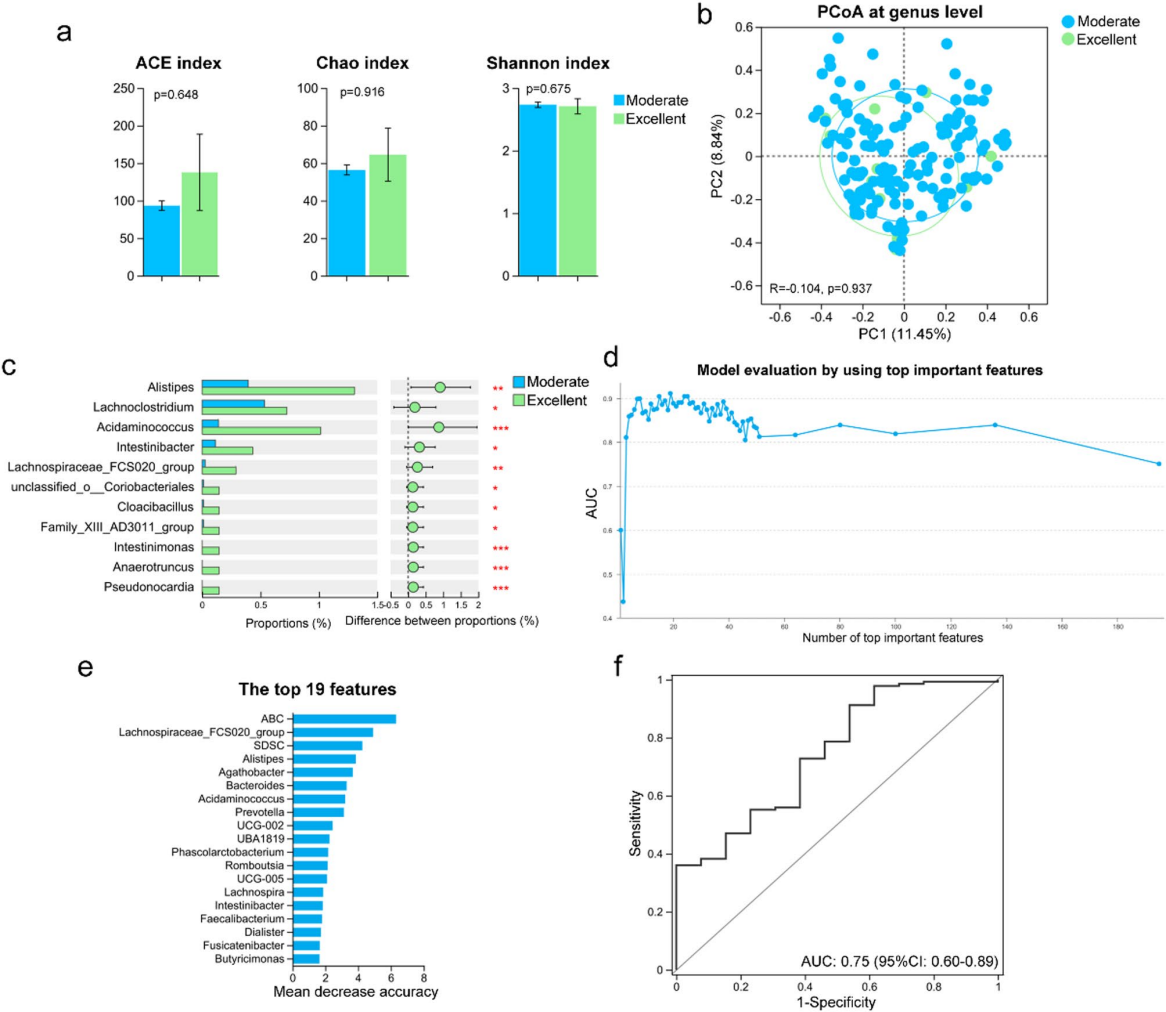
Gut microbiota and clinical symptoms predict the therapeutic efficacy of washed microbiota transplantation (WMT) in children with autism spectrum disorders (ASD). (**a**) Alpha diversity indices (Ace, Chao, Shannon) of gut microbiota in children with ASD showing excellent (*n* = 13 treatment courses) vs. moderate (*n* = 148 treatment courses) improvement after WMT. (**b**) PCoA illustrating gut microbiota composition differences between the two response groups. (**c**) Relative abundances of differentially enriched genera between children with excellent and moderate improvement. (**d**) Area Under the Curve (AUC) values of predictive models based on varying numbers of top-ranked features. (**e**) Top 19 important features, including gut microbial taxa and clinical scores, identified by the random forest model. (**f**) Receiver Operating Characteristic (ROC) curve distinguishing excellent vs. moderate responders using the top 19 features. *p < 0.05; **p < 0.01; ***p < 0.001

Extensive research has established a relationship between pre-treatment clinical features and gut microbiota composition with the efficacy of FMT [34]. In this context, the gut microbiota of children with ASD who achieved excellent improvements post-WMT (with reductions in both ABC and CARS scores ranking in the top 25% compared to before this WMT treatment) versus those with moderate responses was compared. Among all treatments, 148 WMT courses resulted in moderate responses, while 13 courses led to excellent improvements. Alpha and beta diversity analyses revealed no significant differences in the richness, diversity, or structure of the gut microbiota between children with excellent and moderate responses to WMT (Fig. 7a-b). However, the pre-treatment abundance of bacteria such as *Alistipes*, *Acidaminococcus*, and *Intestinimonas* was significantly higher in children with excellent WMT responses (Fig. 7c).

Additionally, a predictive model for WMT efficacy in ASD was developed using both pre-treatment gut microbiota and clinical scales. Random forest analysis revealed that a model incorporating the top 19 most significant gut bacteria and clinical scales from children with ASD demonstrated optimal performance (Fig. 7d). These top 19 bacteria and clinical scales are depicted in Fig. 7e. The ROC curve demonstrated that this model effectively distinguished between the treatment outcomes of WMT in ASD, with an area under the curve (AUC) of 0.75 (Fig. 7f). These results suggest that pre-treatment gut microbiota characteristics are linked to the clinical efficacy of WMT in ASD and that a model integrating gut microbiota and clinical scales can reliably predict WMT outcomes.

### Tongue-coating microbiota, combined with clinical symptoms, can predict WMT efficacy with results comparable to gut microbiota

Although the gut microbiota can predict the effectiveness of WMT in treating ASD, the inconvenience of stool sampling necessitates the identification of alternative, easily accessible indicators linked to the gut microbiota for constructing a predictive model for WMT efficacy. In this study, the abundance of specific tongue-coating bacteria, such as *Streptococcus*, *Lachnoclostridium*, and *Collinsella*, in ASD mice post-FMT was positively correlated with their abundance in the gut (Additional file 5: Figure S5). Furthermore, following WMT, the tongue-coating microbiota of children with ASD correlated with several key gut bacteria, including *Lachnospira*, *Phascolarctobacterium*, and *Dialister*, which are crucial for predicting WMT efficacy (Additional file 6: Figure S6). These results suggest that the tongue-coating microbiota may also serve as a predictor for WMT outcomes.

**Fig. 8:**
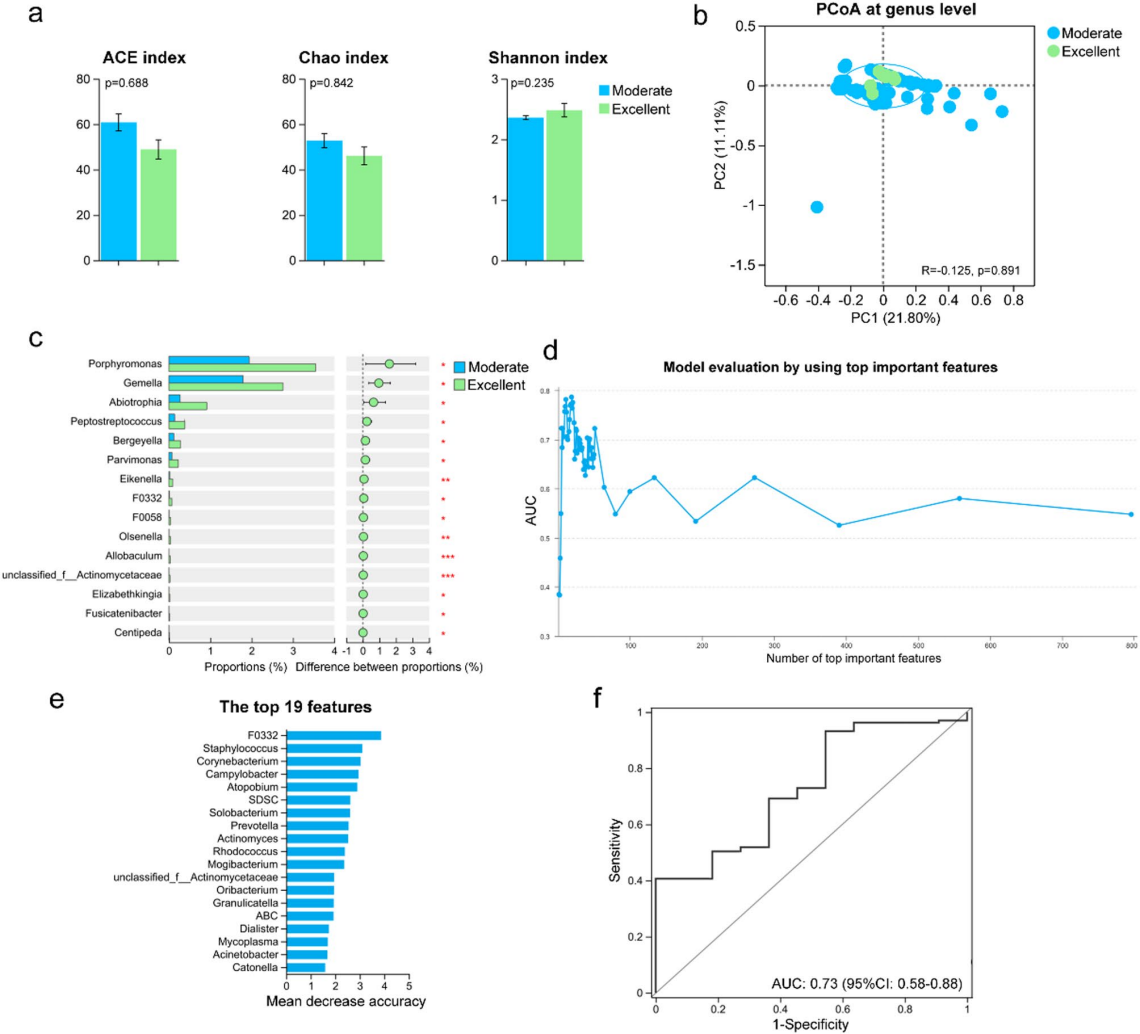
Predictive accuracy of the therapeutic efficacy of WMT in children with ASD, based on tongue-coating microbiota, is comparable to that of gut microbiota. (**a**) Alpha diversity indices (Ace, Chao, Shannon) of tongue-coating microbiota in children with ASD showing excellent (*n* = 11 treatment courses) vs. moderate (*n* = 136 treatment courses) improvement after WMT. (**b**) PCoA illustrating differences in tongue-coating microbiota between the two response groups. (**c**) Relative abundances of differentially enriched genera between children with excellent and moderate improvement. (**d**) Area under the curve (AUC) values of predictive models based on varying numbers of top-ranked features. (**e**) Top 19 important features, including tongue-coating microbial taxa and clinical scores, identified by the random forest model. (**f**) Receiver operating characteristic (ROC) curve distinguishing excellent vs. moderate responders using the top 19 features. *p < 0.05; **p < 0.01; ***p < 0.001

To test this hypothesis, the tongue-coating microbiota of children with ASD who showed excellent improvements post-WMT was compared with those who had moderate responses. Additionally, a predictive model utilizing pre-treatment tongue-coating microbiota and clinical scales was developed to assess WMT efficacy in ASD. Alpha and beta diversity analyses showed no significant differences in the richness, diversity, or structure of the tongue-coating microbiota between children with excellent and moderate responses to WMT (Fig. 8a-b). Genus-level abundance analysis revealed a significant increase in bacteria such as *Porphyromonas*, *Gemella*, and *Abiotrophia* in the tongue coatings of children who experienced excellent improvements post-WMT (Fig. 8c). Random forest analysis identified the best predictive model for treatment efficacy, constructed using the top 19 important features, including tongue-coating microbiota and clinical scales (Fig. 8d-e). Similar to models combining gut microbiota and clinical scales, the model incorporating tongue-coating microbiota and clinical scales also demonstrated good predictive power for WMT treatment efficacy in children with ASD (AUC = 0.73; Fig. 8f). These results indicate the correlation between the gut and tongue-coating microbiota post-WMT and validate the tongue-coating microbiota’s effectiveness in predicting WMT outcomes for ASD, paralleling the predictive capabilities of gut microbiota.

## Discussion

**Fig. 9:**
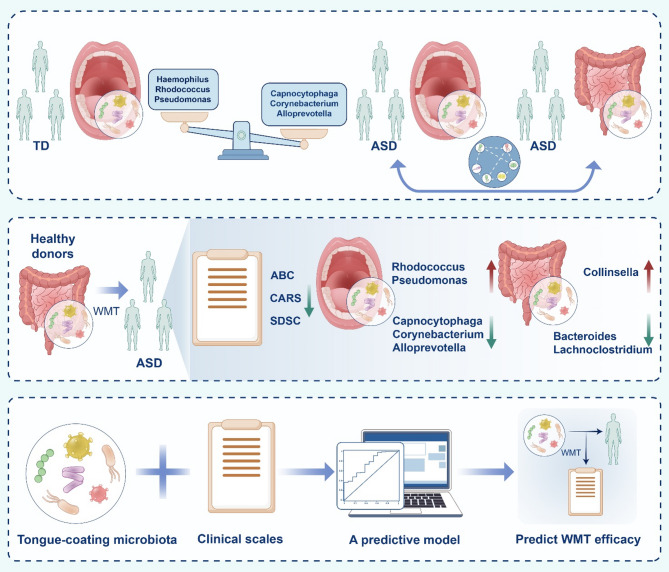
The tongue-coating microbiota of children with ASD exhibited significant changes and was linked to both gut microbiota and clinical symptoms. WMT improved autism symptoms in children with ASD, significantly affecting the characteristics of both the tongue-coating and gut microbiota. Additionally, combining tongue-coating microbiota with clinical characteristics before treatment can predict the effectiveness of WMT for ASD. ABC, Aberrant Behavior Checklist; ASD, Autism Spectrum Disorders; CARS, Childhood Autism Rating Scale; SDSC, Sleep Disturbance Scale for Children; TD, Typically Developing; WMT, Washed Microbiota Transplantation

This study revealed significant alterations in the tongue-coating microbiota of children with ASD, closely associated with both gut microbiota and clinical symptoms. The transplantation of healthy gut microbiota improved autism-like behaviors in both mice and children while also modulating the tongue-coating microbiota. Additionally, integrating pre-treatment tongue-coating microbiota with clinical characteristics enabled precise prediction of WMT efficacy, with performance comparable to gut microbiota-based models (Fig. 9).

Although the oral microbiota in ASD has been extensively studied, most research has focused on saliva. Two saliva-based studies and one investigating tongue-coating microbiota found no significant differences in alpha and beta diversity between children with ASD and healthy controls [17, 18, 35]. However, Hicks et al. observed that while salivary richness was similar, the microbial structure differed significantly between ASD and TD children [36]. Unlike saliva, which reflects a composite microbiome from multiple oral niches, tongue-coating microbiota is more stable, less affected by transient factors (e.g., food), and strongly associated with the dorsal oral mucosa [37]. This study demonstrated that children with ASD exhibited significantly higher richness and distinct microbial structures in the tongue coating compared to TD children. Notably, *Haemophilus* abundance was reduced and negatively correlated with CARS scores, while *Corynebacterium* was elevated and positively correlated, suggesting these taxa as potential biomarkers for ASD severity. Functional predictions highlighted enhanced chemoheterotrophy and fermentation pathways. These findings suggest that the tongue-coating microbiota in ASD undergoes substantial structural and functional changes, which may contribute to disease progression. However, confounding factors—such as oral hygiene, dietary habits, and dental status—pose challenges in controlling, highlighting the need for further validation.

While numerous studies have highlighted a connection between the oral and gut microbiota [38–40], this relationship has rarely been explored in patients with ASD. A study of 19 ASD patients found that only *Escherichia* and an unspecified genus of *Clostridiales* co-occurred with saliva genera [18]. In contrast, the present study, with a larger sample size, identified a more complex and extensive oral–gut microbial network in children with ASD. Notably, several tongue-coating genera linked to clinical symptoms, including *Capnocytophaga*, *Delftia*, and *Corynebacterium*, co-occurred with gut taxa such as *Phascolarctobacterium*, *ParaBacteroides*, and *Escherichia/Shigella*. These findings suggest that oral–gut microbial interactions may contribute to the pathogenesis or progression of ASD.

Sharon et al. and prior studies have shown that transplanting gut microbiota from individuals with ASD into germ-free mice induces core autistic-like behaviors [7, 33]. In contrast, the present study demonstrated that transferring healthy gut microbiota into germ-free ASD mice alleviated behavioral symptoms, reduced neuroinflammation, and improved intestinal morphology—key features linked to ASD progression [41]—compared to mice receiving ASD-derived microbiota. Furthermore, mice receiving healthy microbiota exhibited increased microbial richness and diversity, along with enrichment of beneficial bacterial taxa. These findings suggest that healthy gut microbiota transplantation may effectively alleviate ASD-related symptoms by reshaping gut microbial composition.

Consistent with previous studies showing that FMT can reshape gut microbiota and alleviate ASD symptoms [9–11], this study found that WMT induced substantial structural changes in the gut microbiota of children with ASD, accompanied by significant clinical improvements. Notably, the abundances of *Bacteroides* and *Lachnoclostridium*—both associated with ASD severity—were significantly reduced after WMT, suggesting a potential role in ASD treatment. In line with earlier reports linking FMT efficacy to baseline gut microbiota [10], higher pre-treatment levels of *Alistipes*, *Acidaminococcus*, and *Intestinimonas* were associated with improved WMT outcomes. Given the correlation between FMT efficacy, pre-treatment clinical characteristics, and microbial profiles [34], a predictive model incorporating both pre-treatment gut microbiota and clinical features was developed, demonstrating strong performance. This approach not only supports precision therapy but could also inform donor selection by matching microbial profiles, optimizing therapeutic outcomes.

Given the well-established link between oral and gut microbiota [38–40] and the observed correlation between tongue-coating and gut microbiota in children with ASD, this study further investigated the impact of healthy gut microbiota transplantation on the tongue-coating microbiota in ASD mice. Compared to mice receiving ASD-derived microbiota, those transplanted with healthy microbiota exhibited significant structural shifts and compositional changes in the tongue-coating microbiota. Several taxa in the tongue coating were positively correlated with their gut counterparts. Similarly, in children with ASD, WMT markedly altered the tongue-coating microbiota, making it more similar to that of TD children. Notably, the abundance of *Haemophilus*—which is negatively associated with ASD severity—and functional pathways related to chemoheterotrophy and fermentation were significantly reduced after WMT, suggesting their involvement in ASD pathology. Emerging evidence indicates that oral microbiota may influence ASD progression by promoting systemic inflammation, disrupting the blood–brain barrier, migrating to the brain, and modulating prefrontal cortex gene expression [19, 42–44]. Additionally, oral microbiota transplantation has been shown to reshape gut microbial composition in ASD mice [19], raising the possibility that tongue-coating microbiota may influence gut colonization and, consequently, the efficacy of WMT. These findings highlight the potential role of oral–gut microbial interactions in ASD treatment, although the mechanisms through which gut microbiota may regulate oral microbial communities require further investigation.

Combining pre-treatment gut microbiota with clinical characteristics can predict the efficacy of WMT in treating ASD. However, obtaining fecal samples from patients is often challenging, particularly since many children with ASD experience constipation [45], which can make pre-treatment fecal sampling difficult. Given the significant correlation between the gut and tongue-coating microbiota in children post-WMT and existing evidence that oral microbiota can also predict treatment outcomes [20, 21], an alternative approach was explored. Tongue-coating microbiota, which is easier to collect than fecal samples, was used to construct a predictive model for WMT efficacy incorporating both pre-treatment microbiota and clinical features. The predictive performance of this model was comparable to those based on gut microbiota and clinical characteristics. These findings underscore the potential of using a more accessible model to identify which children with ASD are most likely to benefit from WMT treatment.

This study has several limitations. First, the absence of metagenomic sequencing restricted the species-level resolution of microbial changes following WMT, preventing the development of species-level predictive models. Second, dietary data post-WMT were not collected, limiting the ability to assess how diet might influence the correlation between oral and gut microbiota, despite evidence that both are shaped by dietary factors. Third, the findings are correlational in nature; although changes in tongue-coating microbiota were associated with gut microbiota alterations and treatment outcomes, causal relationships cannot be inferred. Functional studies, such as gnotobiotic transplantation of oral microbiota, are needed to establish causality. Fourth, there was a gender imbalance in the cohort (~ 94% male). While this reflects the higher prevalence of ASD in males, it may nonetheless impact microbiota composition and treatment response. Finally, an age mismatch exists between the animal model and the clinical population. Eight-week-old mice, which developmentally approximate 14-year-old humans [46], were compared with children whose average age was approximately 6 years, potentially limiting the translational relevance.

## Conclusions

In conclusion, this study revealed correlations among the tongue-coating microbiota, gut microbiota, and disease severity in children with ASD. Moreover, WMT not only addresses ASD symptoms but also significantly alters the tongue-coating microbiota profiles in these children. Additionally, a model incorporating pre-treatment tongue-coating microbiota and clinical characteristics effectively predicts the therapeutic efficacy of WMT for ASD.

## Electronic supplementary material

Below is the link to the electronic supplementary material.

Supplementary Material 1

Supplementary Material 2

Supplementary Material 3

Supplementary Material 4

Supplementary Material 5

Supplementary Material 6

Supplementary Material 7

Supplementary Material 8

## Data Availability

The datasets generated and/or analysed during the current study are available in https://www.ncbi.nlm.nih.gov/bioproject/PRJNA1116522, reference number PRJNA1116522, and within the article and its supplementary materials.

## Abbreviations

ASD: Autism spectrum disorder
FMT: Fecal microbiota transplantation
TD: Typically developing
WMT: Washed microbiota transplantation
CARS: Childhood Autism Rating Scale
ABC: Aberrant Behavior Checklist
SDSC: Sleep Disturbance Scale for Children
PBMC: Peripheral blood mononuclear cell
ASVs: Amplicon sequence variants
PCoA: Principal coordinate analysis
AUC: Area Under the Curve
