# Supplementary figures and images for: Tongue-coating microbiota as a predictive biomarker of washed microbiota transplantation efficacy in pediatric autism: integration with clinical features
Source: J Transl Med. 2025 Jul 16;23:799. doi: 10.1186/s12967-025-06846-z (PMC12269187; doi:10.1186/s12967-025-06846-z)

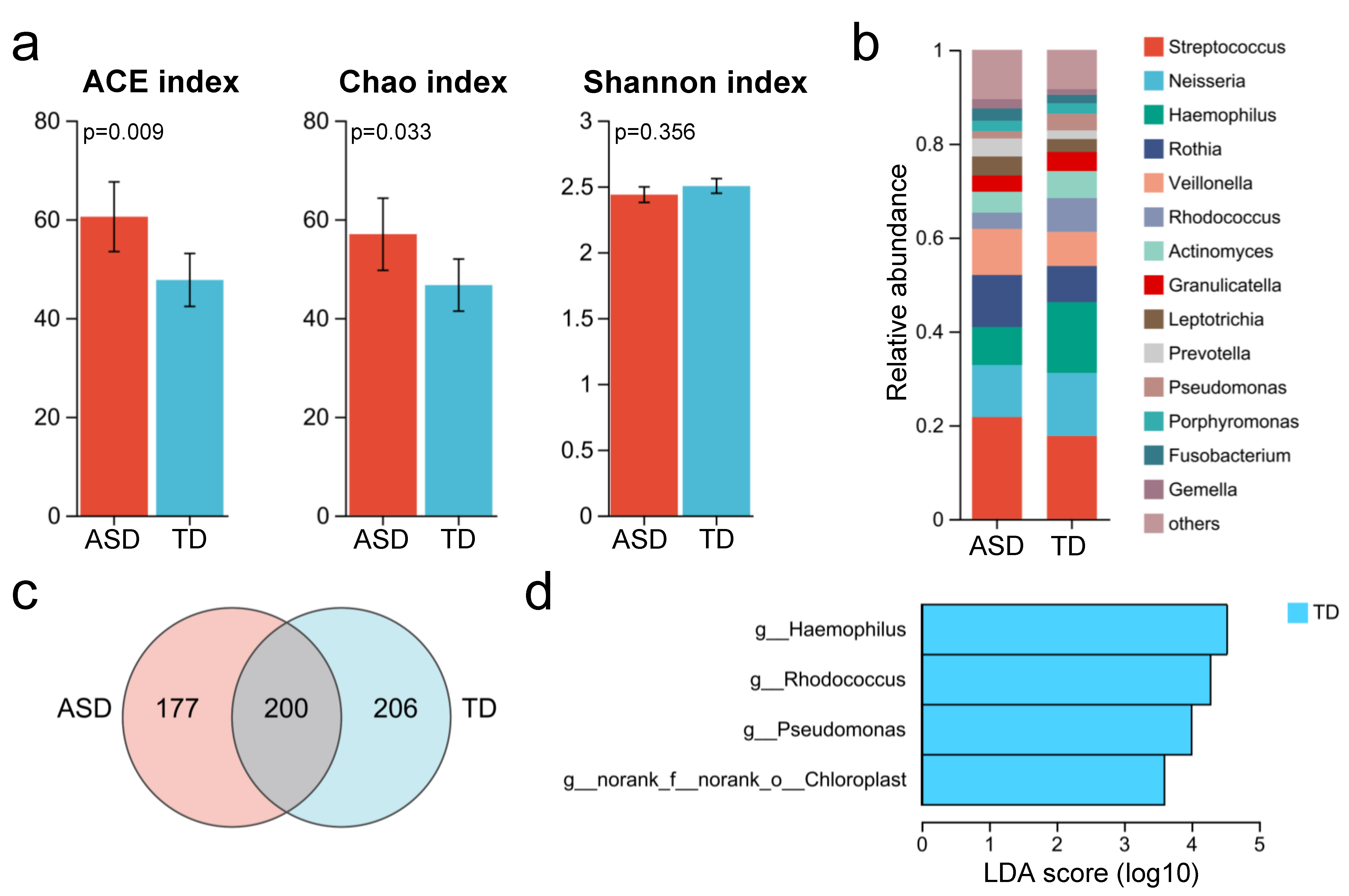

Supplement: Supplementary file 1 — Supplementary Material 1 [file 12967_2025_6846_MOESM1_ESM.tif]

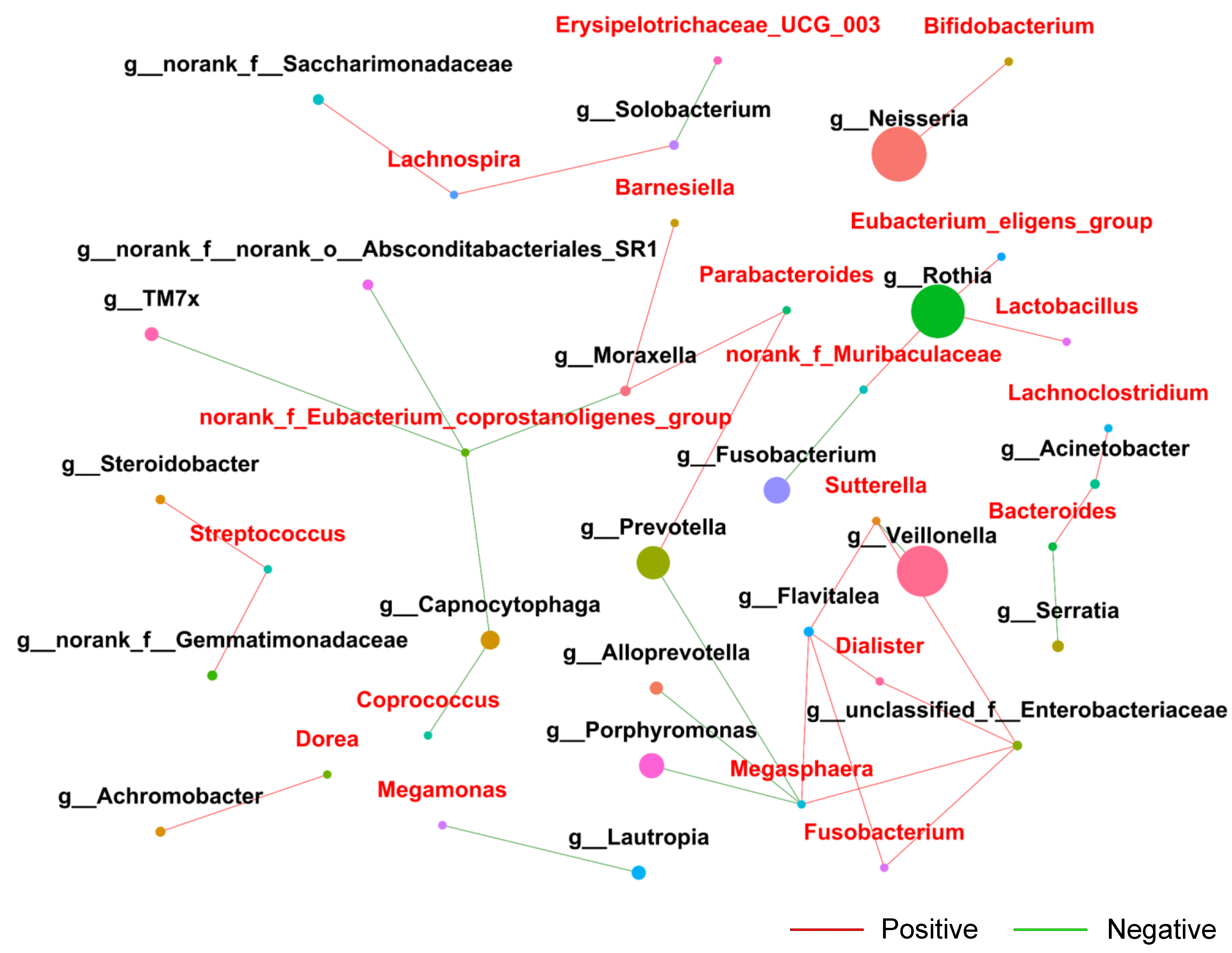

Supplement: Supplementary file 2 — Supplementary Material 2 [file 12967_2025_6846_MOESM2_ESM.tif]

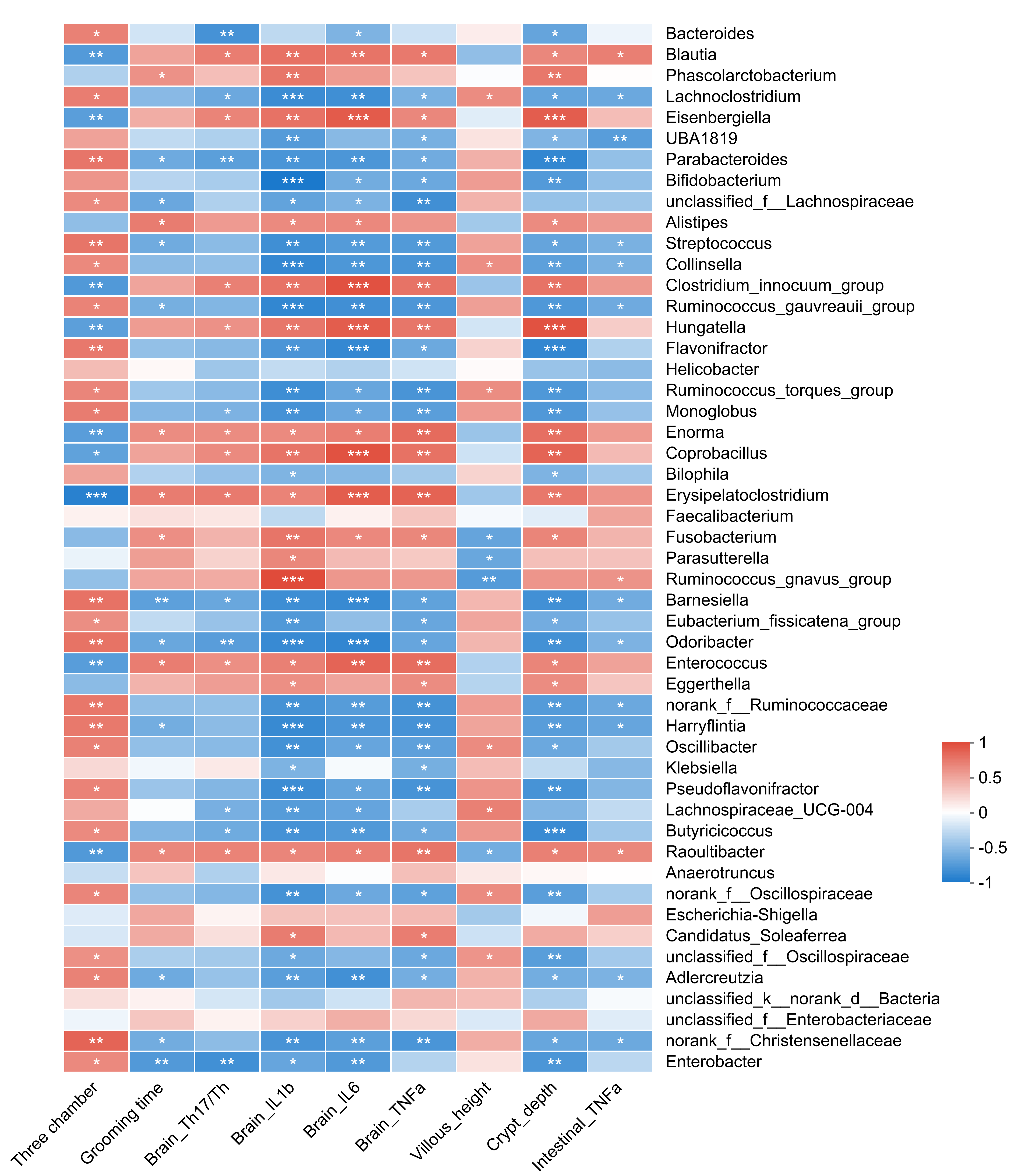

Supplement: Supplementary file 3 — Supplementary Material 3 [file 12967_2025_6846_MOESM3_ESM.tiff]

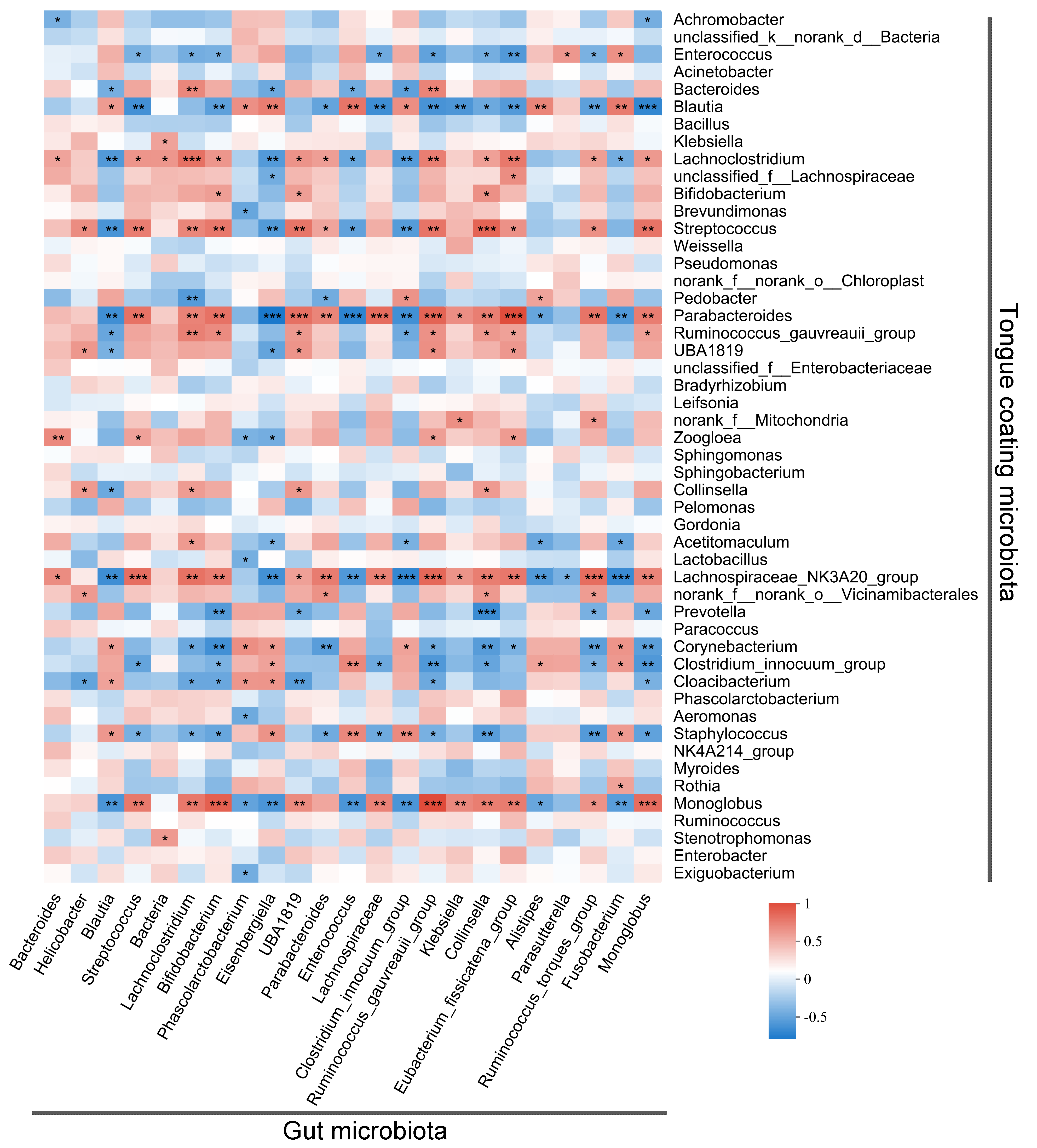

Supplement: Supplementary file 5 — Supplementary Material 5 [file 12967_2025_6846_MOESM5_ESM.tif]

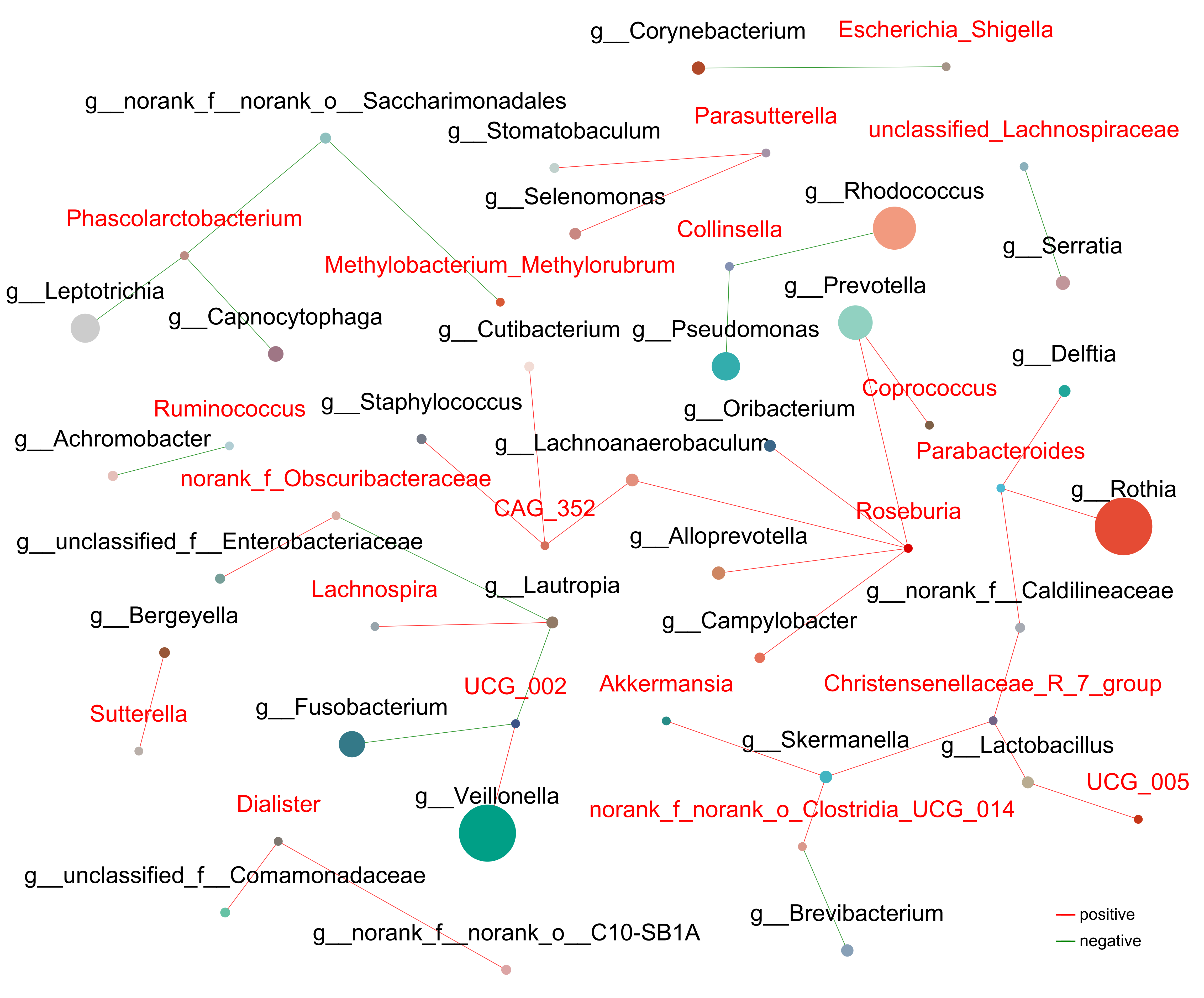

Supplement: Supplementary file 6 — Supplementary Material 6 [file 12967_2025_6846_MOESM6_ESM.tif]
