# Supplementary material for: Tongue-coating microbiota as a predictive biomarker of washed microbiota transplantation efficacy in pediatric autism: integration with clinical features
Source: J Transl Med. 2025 Jul 16;23:799. doi: 10.1186/s12967-025-06846-z (PMC12269187; doi:10.1186/s12967-025-06846-z)
Supplement: Supplementary file 7 — Supplementary Material 7 [file 12967_2025_6846_MOESM7_ESM.docx]

**Table S1. Baseline characteristics of children with autism spectrum disorder (ASD) undergoing washed microbiota transplantation (WMT) treatment.**

|  | Children with ASD (n=83) |
| --- | --- |
| Age (years) | 6.00 (4.00–8.00) |
| Male (%) | 73 (87.95) |
| BMI (kg/m^2^) | 16.45 (14.92–18.27) (n=81) |
| Duration (years) | 3.18 (2.03–5.09) |
| ABC | 63.50 (49.75–76.00) (n=82) |
| CARS | 36.00 (34.00–38.00) (n=81) |
| SDSC | 50.00 (42.00–59.50) (n=81) |

Data are presented as the mean ± standard deviation or n (%). ABC, Aberrant Behavior Checklist; ASD, autism spectrum disorder; BMI, body mass index; CARS, Childhood Autism Rating Scale; SDSC, Sleep Disturbance Scale for Children; WMT, washed microbiota transplantation.
